# Exploring BCG to deliver avidin fusion antigens from *Schistosoma mansoni*

**DOI:** 10.1590/0074-02760240167

**Published:** 2025-03-03

**Authors:** Lais Sayuri Yamamoto, Monalisa Martins Trentini, Dunia Rodriguez, Paulo Henrique Santana Silveira, Arthur Daniel Januzzi, Ana Carolina de Oliveira Carvalho, Luciana Cezar de Cerqueira Leite, Alex Issamu Kanno

**Affiliations:** 1 Instituto Butantan, Laboratório de Desenvolvimento de Vacinas, São Paulo, SP, Brasil; 2 Universidade de São Paulo, Programa de Pós-Graduação Interunidades em Biotecnologia, São Paulo, SP, Brasil

**Keywords:** BCG, avidin-biotin, rhizavidin, vaccine development, Schistosoma mansoni

## Abstract

**BACKGROUND:**

Bacillus Calmette-Guérin (BCG) is one of the most successful vaccines in the world and evidence suggests it can be used as a bacterial vector to deliver heterologous antigens.

**OBJECTIVES:**

We evaluated whether BCG could be biotinylated and used as a carrier of *Schistosoma mansoni* antigen tetraspanin-2 (TSP-2) fused with rhizavidin, an avidin analog.

**METHODS:**

BCG was grown and biotinylated. The recombinant protein Rzv:TSP-2 was produced and purified from *Escherichia coli*. The biotinylation and antigen coupling was analysed by flow cytometry, enzyme-linked immunosorbent assay (ELISA) and Western blot. Vaccine immunogenicity was tested in immunised mice by the assessment of lung and splenic T cells.

**FINDINGS:**

BCG can be biotinylated, which in turn, can be coupled with Rzv:TSP-2. After a series of optimisations which involved molarity of the biotin, ratio of BCG:reagent and the concentration of Rzv:TSP-2 used, almost 50% of the bacteria were biotinylated and 35% coupled with antigen. Although a clear adjuvant effect of BCG was observed, evaluation of immune response in immunised mice demonstrated an overall low immunogenicity of the BCG-Rzv:TSP-2.

**MAIN CONCLUSION:**

These results demonstrated the use of BCG as a carrier of avidin-tagged antigens. Further optimisations are needed in order to strengthen the stability of tagged proteins in order to produce antigen-specific immune responses.

Vaccines are one of the most important accomplishments of the modern medicine. They represent a milestone in human health and prevent millions of deaths each year. Through the history of vaccine development several technologies and platforms arise and sustained the continuous application of vaccines to a wide range of pathogens.^1^ ^,^ ^2^ The emergence of coronavirus disease 2019 (COVID-19) supported the expansion of mRNA and viral-vectored technologies among others. The emergence of new infectious diseases and the persistence of others indicates that innovative and new approaches need to be constantly investigated.^3^ ^,^ ^4^

The idea of using bacillus Calmette-Guérin (BCG) to fight Schistosomiasis, a parasitic infectious disease, is partially based on BCG’s tropism for the lung compartment.^5^ Along with its powerful activation of systemic immune responses it can also stimulate the lungs in important innate and adaptative immune mechanisms.^6^ ^,^ ^7^ ^,^ ^8^ For *Schistosoma mansoni*, this may play an important role since the lungs represent the main attrition site to the parasite’s migration before it can reach the portal veins and mature to adult worms. The induction of antigen-specific immune responses in this organ could prevent its migration and, as a consequence, inhibit its maturation.^9^ ^,^ ^10^

BCG is a centenary vaccine and it is estimated that more than 3 billion people around the world were immunised. It is an attenuated strain of *Mycobacterium bovis* that required almost 13 years and more than 230 *in vitro* passages which after became avirulent in animals but capable of conferring cross-protection against *M. tuberculosis*.^11^ BCG itself is a powerful adjuvant and many have combined BCG or its immunogens to enhance the immunogenicity against other antigens. We and others have explored BCG as a vector to express and deliver heterologous antigens.^12^ ^,^ ^13^ ^,^ ^14^ ^,^ ^15^ ^,^ ^16^ In this scenario, many bacterial and viral antigens were expressed as recombinant BCG strains, reviewed in.^17^ Previous attempts to express *S. mansoni* antigens in recombinant BCG proved to be difficult. Of the many vaccine candidates tested for *S. mansoni*, the tetraspanin-2 (TSP-2) antigen induced one of the highest protection levels observed in the mouse model. The immunisation with recombinant TSP-2 combined with Freund’s adjuvant induced a 53% reduction in adult worms and 61% decrease in oviposition.^18^ At the same time, previous work from our lab also demonstrates the suitability of exploiting the avidin-biotin natural affinity to combine strong adjuvants and antigens of interest.^19^ In this work we explored the use of BCG as a biotinylated carrier to the *S. mansoni* antigen TSP-2 in fusion with rhizavidin, a streptavidin analog.

## MATERIALS AND METHODS

*Bacterial strains, media and culture conditions* - *Escherichia coli* BL21 (DE3) strain (Thermo Scientific) transformed with pET21b-Rzv:TSP-2 was used to express the recombinant protein. Bacteria was grown in terrific broth (TB) added with ampicillin (100 µg/mL) and cultured at 37ºC under agitation 250 rpm (Gyromax 774R, Amerex Instruments) using Tunair full-baffled flasks (Merck). BCG was grown in Middlebrook 7H9 supplemented with oleic acid-albumin-dextrose-catalase (OADC, BD), Tween 80 0.05% and glycerol 0.5% at 37ºC and 5% CO_2_. BCG was resuspended in glycerol 10% and aliquots maintained at -80ºC until use. Middlebrook 7H10 supplemented with OADC was used to determine the viability of BCG by counting colony forming units (CFU).

*Expression and purification of Rzv:TSP-2* - Transformed *E. coli* was grown in 800 ml of TB containing ampicillin at 37ºC under agitation 250 rpm. At ~ optical density (OD)_600_ 0.6 or 3, cultures were acclimatised to 18ºC for 30 min, 1 mM of IPTG (Sigma-Aldrich) was added and the culture incubated for up to 18 h. At regular intervals an aliquot was removed to determine growth and Rzv:TSP-2 soluble production. After expression, the culture was centrifuged at 4ºC, 10 min, 4,000 rpm (Eppendorf Centrifuge 5810 R), the bacterial pellet resuspended in lysis buffer (150 mM Tris-HCl pH 8; 300 mM NaCl, 1 mM PMSF) and mechanically lysed using GEA homogeniser PandaPLUS 1000. After lysis, the sample was centrifuged at 4ºC, 30 min, 12,000 rpm at a High-speed Refrigerated Centrifuge (Hitachi) to separate soluble and insoluble fractions. Soluble fraction was applied to HisTrap FF 5 mL (Cytiva) column using the AKTA Start equipment (GE). Histidine-tagged protein was eluted using an imidazole (Sigma-Aldrich) gradient (0-300 mM). Protein samples were stored at 4ºC and analysed by sodium dodecyl sulphate-polyacrylamide gel electrophoresis (SDS-PAGE) and Western blot.

**Fig. 1:**
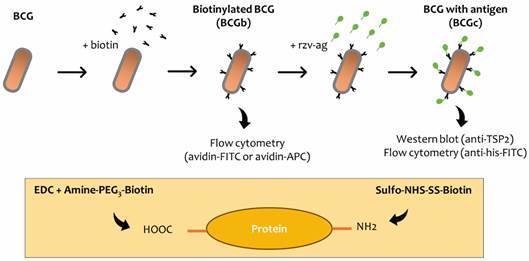
schematic representation of the strategy for biotinylation and antigen coupling to bacillus Calmette-Guérin (BCG). BCG was biotinylated (BCGb). Antigen in fusion with rhizavidin (rzv-ag) are incubated with biotinylated BCG to bind the antigens onto BCG’s surface (BCGc). The efficacy of biotinylation and antigen coupling were evaluated by flow cytometry and western blot (using avidin-FITC, avidin-APC, anti-his-FITC or anti-TSP2). Different reagents were used to biotinylate (yellow box). A combination of EDC ((3-dimethylaminopropyl)carbodiimide) and Amine-PEG_3_-Biotin with and without the stabilisation provided by Sulfo-NHS. This reagent will form an amide bond with reactive carboxyl groups. Sulfo-NHS-SS-Biotin reacts with deprotonated amine groups.

*Biotinylation of BCG cell surface* - Aliquots of BCG substrain Danish were washed in phosphate-buffered saline (PBS) with Tween 20 0.05% (PBS-T) and resuspended in PBS. Immediately before use 50 mM of EZ-link Amine-PEG_3_-Biotin (Thermo Fisher) and 100 mM of carbodiimide (EDC) (Thermo Fisher) were diluted in PBS and filtered through at 0.22 µm (Millipore). BCG was incubated with 0.3 µmol of EDC and 3 µmol of Amine-PEG_3_-Biotin for 2 h in room temperature (RT). Samples were washed with PBS-T and resuspended in PBS-T. Additionally, BCG was also biotinylated using an alternative reagent without the requirement of EDC. Briefly, 10 mM of Sulfo-NHS-SS-Biotin (Thermo Fisher) was resuspended in filtered-sterile water and BCG incubated using a range of concentrations (4 mM to 8 nM), temperatures (4 - 23ºC) and period (0.5 - 4 h).^20^ Samples were washed with PBS-T and resuspended in PBS-T (Fig. 1).

*Protein endotoxin removal and binding to BCG (BCG-Rzv:TSP-2)* - Protein samples were dialysed using a Slide-A-Lyzer Dialysis Cassettes 3,000 MWCO (Thermo Scientific) in PBS (2 exchanges, 4 h each). Approximately 15-20 mL of protein solution and 2-L of ice-cold PBS. After dialysis, the sample was treated with 1% (v/v) Triton X-114 (Sigma-Aldrich) and incubated at 37ºC for 15 min until phase separation. Next, the mixture was centrifuged using a benchtop centrifuge at 12,000 rpm and the upper phase recovered. This process was repeated twice. To promote the binding of Rzv:TSP-2 and BCG, the recombinant protein was incubated with 10^5^ to 10^8^ CFU of BCG at RT for 1 h. The final product was used in flow cytometry and immunisation experiments.

*Evaluation of biotinylation and protein binding* - Biotinylated BCG was evaluated using avidin-FITC (1:200) or avidin-APC (1:200) (Biolegend). Protein binding was detected using anti-his-FITC (1:200). Fluorochromes were incubated at 4ºC for 40 min and samples fixed in 1% paraformaldehyde (Sigma-Aldrich). Next, samples were analysed using a FACS Canto II flow cytometer (BD). For Western blot analysis, samples were sonicated on ice at an amplitude of 60 Hz for 5 min with 1 s pulses (Ultrasonic Processor GE 100). Protein extracts were then separated on SDS-PAGE using the Mini PROTEAN Tetra Cell system (Bio-Rad, Hercules, CA, USA) and transferred to a PVDF membrane (Merck Millipore, Carrigtwohill, Co. Cork, IRL) using the Trans-Blot Turbo Transfer System (Bio-Rad) (25 V; 1 A; 12 min). Membrane was blocked in PBS-T with 5% skimmed milk and incubated with anti-TSP-2 serum (1:2,000) (raised in-house in immunised mice) at room temperature for 1 h. Next, the secondary antibody (goat anti-mouse peroxidase-conjugated) (Sigma-Aldrich) was incubated for 1 h (1:3,000). The reaction was visualised using the ECL Prime according to the manufacturer’s instructions (Cytiva-Amersham). Images were captured using the ImageQuant LAS 4000 system (GE Healthcare Life Sciences). To evaluate the rhizavidin’s ability to bind biotin, a 96-well enzyme-linked immunosorbent assay (ELISA) plate was sensitised with a biotinylated protein (1 µg/mL). Briefly, the plate was incubated with the protein for 16-18 h at RT and blocked using 10% skimmed milk (Molico) for 30 min at 37ºC. Plates were washed three times using PBS containing 0.05% Tween 20 and incubated with serial dilutions of equivalent molar concentrations of either Rzv:TSP-2 or TSP-2 (without rhizavidin) and incubated for 1 h at RT. Binding of Rzv:TSP-2 or TSP-2 was detected using anti-his-HRP (1:1,000) (Sigma-Aldrich) incubated for 30 min. Reaction was revealed using TMB OptEIA (BD) as substrate and hydrogen peroxide (Sigma-Aldrich). Reaction was stopped using 4 M H_2_SO_4_ (Sigma-Aldrich) and the absorbance read at 492 nm using a microplate reader EPOCH (BioTek Instruments).

*Immunisation and immune response induced by BCG-Rzv:TSP-2* - Groups of female BALB/c mice (n = 5/group) were immunised subcutaneously either a single dose of Rzv:TSP-2 (protein alone, 10 µg), BCG (10^6^ CFU), BCG mixed with Rzv:TSP-2 (10^6^ CFU + 10 µg protein) and BCG-Rzv:TSP-2 (100 µL of the final product). Thirty days after immunisation lungs and spleens were recovered and single cells stimulated with 10 µg/mL of TSP-2 for 4-6 h at 37ºC and 5% CO_2_. Monensin (BD) (3 mM) was added and the cells kept under culture for another 12 h. Extracellular markers and intracellular cytokines were detected using: anti: PercP-CD4; PE-Cy7-CD8; APC-Cy7-CD3, FITC-TNF-α; PE-IL-17; APC-IFN-γ and BV421-IL-2 (Bioscience). After washing, cells were acquired by flow cytometry in a FACS Canto II (BD). Data was analysed in FlowJo v10 software.

*Statistical analysis* - The difference between groups was evaluated using Mann-Whitney U-test using GraphPad Prism software. p palues ≤ 0.05 were considered statistically significant.

*Ethics statement* - The animal experiments were approved by the Committee for Ethics in Animal Use at Instituto Butantan (CEUAIB, Protocol 1989150622).

## RESULTS

**Fig. 2:**
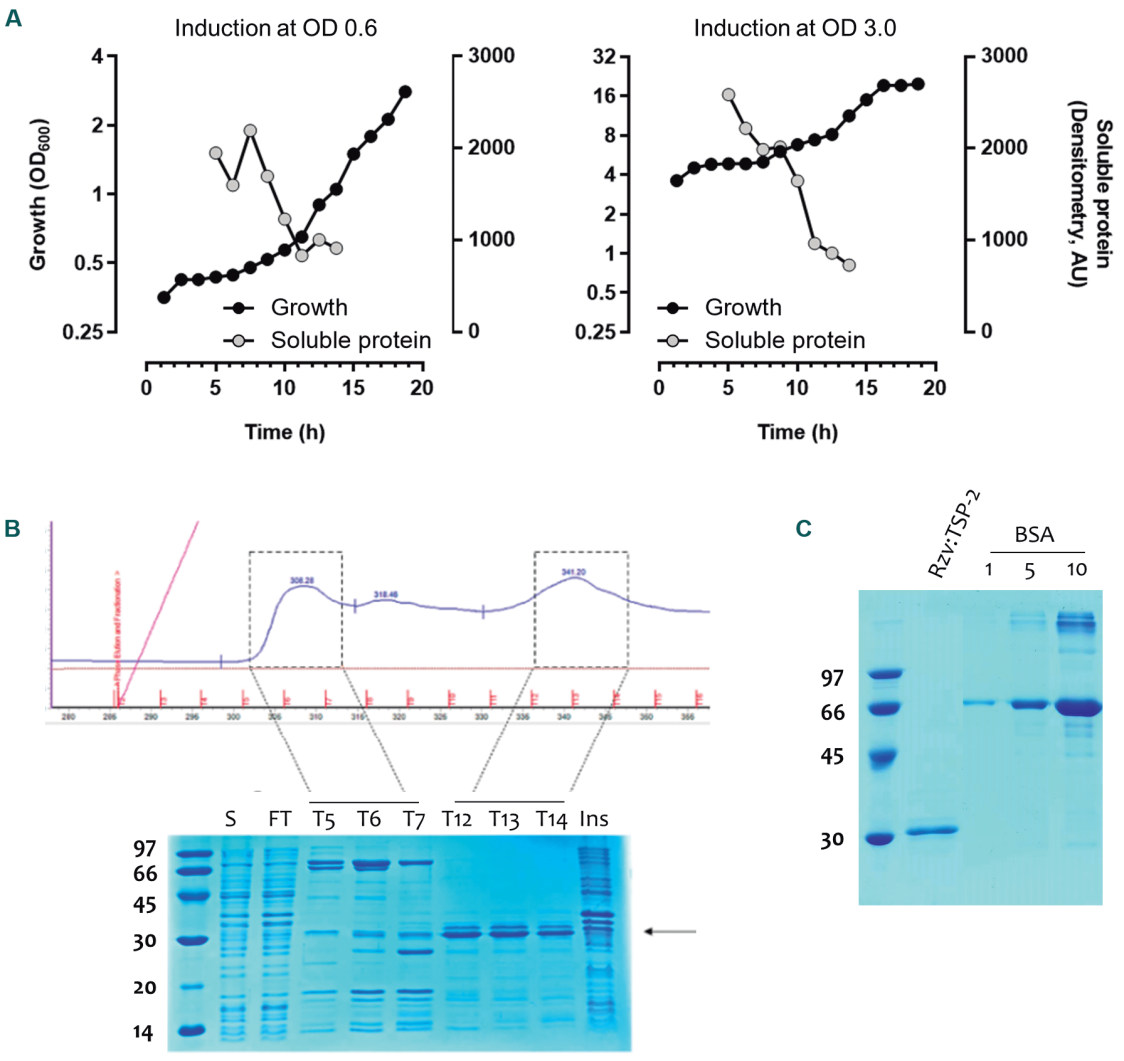
profile of growth and soluble Rzv:TSP-2 expression in *Escherichia coli* BL21 (DE3) after induction. (A) *E. coli* transformed with pET21b containing the *rzv:tsp-2* gene under the T7 promoter regulation was grown in terrific broth (TB) using TunAir full-baffled flasks until the referred optical density (OD) (0.6 or 3.0). Cultures were acclimatised to 18ºC and induced with 1 mM IPTG for up to 18 h under constant agitation. At regular intervals samples were taken to measure OD and soluble Rzv:TSP-2 [determined by densitometry and scaled using arbitrary units (AU) ]. After culture, bacterial pellet was disrupted by mechanical lysis and the soluble fraction separated by immobilised-metal affinity chromatography (IMAC) purification. (B) Elution profile after imizadole gradient elution (0-300 mM). Two distinct peaks (T5-T7 and T12-T14) were applied in sodium dodecyl sulphate-polyacrylamide gel electrophoresis (SDS-PAGE) and stained with Coomassie Blue R250. S: soluble fraction; FT: flowthrough; Ins: insoluble fraction. After purification, fractions T12-T14 were combined and dialysed in phosphate-buffered saline (PBS). (C) 20 µL of the final product was applied in SDS-PAGE and compared to 1, 5 and 10 µg of bovine serum albumin (BSA).

*Expression of Rzv:TSP-2 in recombinant E. coli* - Based on previous knowledge on the expression of soluble Rzv:TSP-2, we tested different temperature of induction and concentration of IPTG. Overall, induction with 1 mM IPTG at 22ºC produced better results in comparison to 37ºC and 0.1 mM IPTG. When the induction OD at induction was evaluated (OD 3.0 and 0.6) profile of soluble Rzv:TSP-2 obtained was similar (Fig. 2A) up to 9 h after induction when it decreases. On the other hand, induction at a higher OD also permit achieving higher ODs (~ 7.0). In comparison, induction at OD 0.6 practically stalled the growth up to 10 h after. After immobilised-metal affinity chromatography (IMAC) purification, the final yield obtained was 170 µg/mL (~ 15 ml) with a purity of ~ 75% (Fig. 2B-C).

**Fig. 3:**
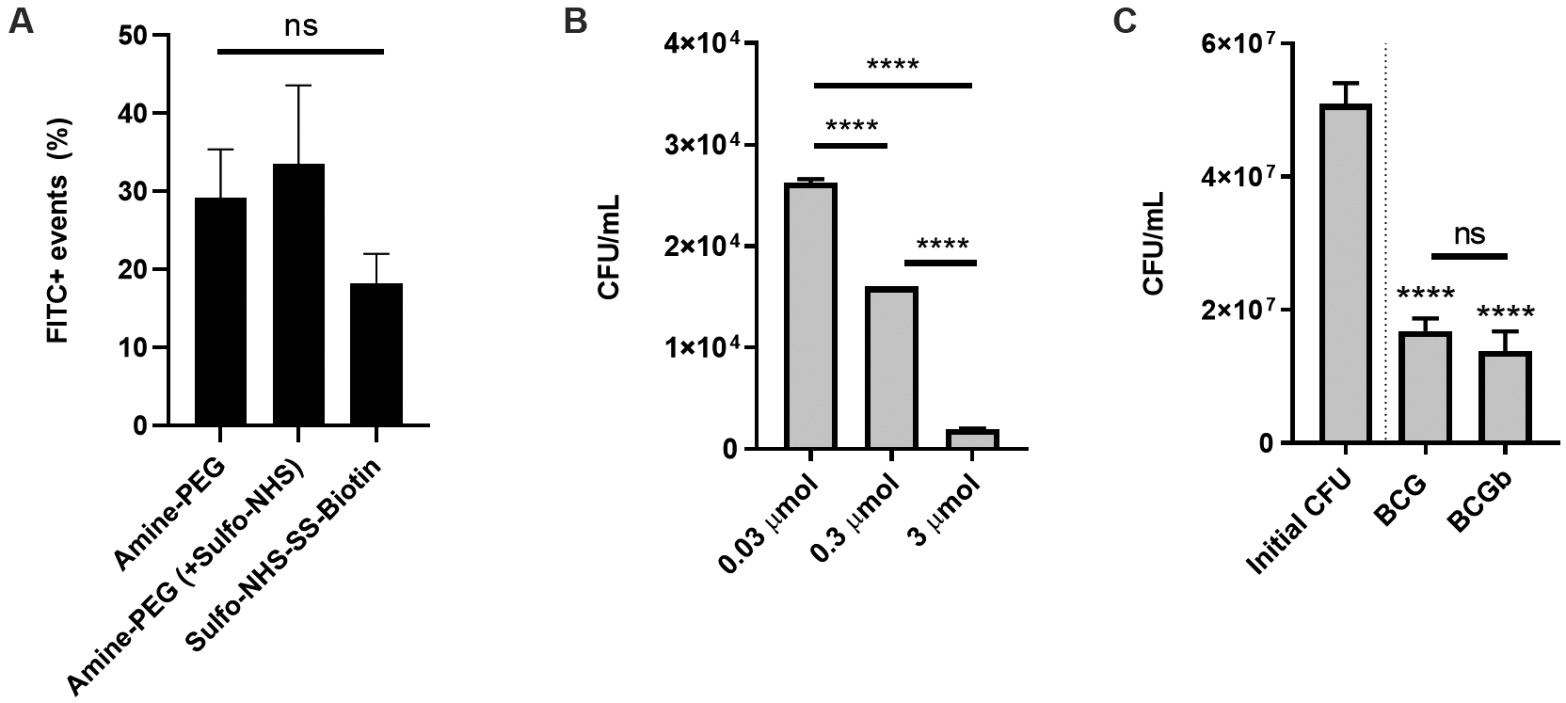
biotinylation and yield of bacillus Calmette-Guérin (BCG) recovered. BCG (n = 3 replicates/condition) was biotinylate using the three different conditions: Amine-PEG, Amine-PEG (+Sulfo-NHS) and Sulfo-NHS-SS-Biotin. (A) After gating BCG (FSC x SSC), FITC positive events were determined based on a non-biotinylated BCG. (B) After biotinylation with different concentrations (0.03 to 3 µmol) of Amine-PEG, BCG was plated onto agar plates to enumerate colony-forming unit (CFU). (C) After biotinylation with Sulfo-NHS-SS-Biotin BCG (BCGb) was plated onto agar plates to enumerate CFU. As comparison the initial aliquot of BCG was plated (initial CFU) and also a BCG that went through the process of washing and incubation but without the reagent (BCG). Difference among groups was determined using one-way analysis of variance (ANOVA) and Tukey’s post-test. p values ≤ 0.05 were considered statistically significant. ****p ≤ 0.0001; ns: not significant.

*Biotinylation of BCG* - In order to evaluate the biotinylation of BCG, three different reactions were conducted. Two of these (PEG_3_ and SPEG_3_) rely on the use of EDC to turn reactive carboxyl groups appropriate for the nucleophile attack by primary amines, e.g. the Amine-PEG_3_-Biotin. The second one adds Sulfo-NHS to improve the intermediate’s stability in water solutions. The third one will react with primary amines in the deprotonated form (hence the use of neutral to slightly basic pH 7-9) (Fig. 3A). When we evaluated the biotinylation of BCG using these methods, SPEG3 provided the highest rate (44%) followed by PEG3 (35%) and Sulfo (23%). On the other hand, evaluation of viability demonstrated a lower recovery of viable cells by the end of the process when using PEG_3_. Sulfo did not decrease the yield of viable cells (Fig. 3B-C). Furthermore, the total recovery was much lower when using PEG_3_ as it requires more steps in the reaction.

**Fig. 4:**
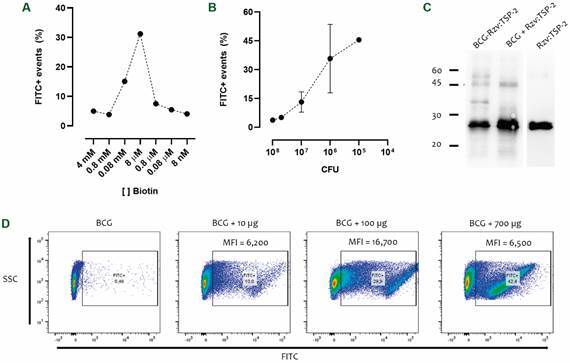
optimisation of biotinylation and antigenic coupling. (A) Bacillus Calmette-Guérin (BCG) was biotinylated with different concentrations of Sulfo-NHS-SS-Biotin (4 mM to 8 nM). (B) Using the optimised biotin concentration, the amount of BCG in the biotinylation reaction was tested [10^8^ to 10^5^ colony-forming unit (CFU) ]. Biotinylated events were detected using avidin-FITC and flow cytometry. (C) Western blot of BCG coupled with Rzv:TSP-2, only mixed with the recombinant protein (BCG + Rzv:TSP-2) and the recombinant protein alone (Rzv:TSP-2). (D) FITC+ events and mean fluorescence intensity (MFI) of BCG after biotinylation and coupling using different concentrations of antigen. Antigen-coupled events were detected using anti-his-FITC and flow cytometry.

*Optimising biotinylation with Sulfo-NHS-SS-Biotin* - In order to improve the number of biotinylated events we investigated the molar ratios of biotin:BCG. At first, from the pre-established condition of 0,8 mM we extended the range from 4 mM to 8 nM. The results demonstrated that the highest biotinylation was achieved when using 8 µM (~ 30%) (Fig. 4A). The amount of BCG used in the reaction was also evaluated after establishing 8 µM as the appropriate concentration of biotin. Ranging the amount of BCG from 10^8^ to 10^5^ CFU in the reaction we observed an inverse dose-response with less BCG resulting in better biotinylation levels. Independent experiments showed that ~ 10^6^-10^5^ CFU as the appropriate amount of BCG (Fig. 4B). Having established a reasonable reaction to generate biotinylated BCG, we investigated the effect of antigen concentration in the generation of BCG-Rzv:TSP-2. Using 10, 100 and 700 µg of Rzv:TSP-2 in the reaction we observed a dose-response with 9, 30 and 37% of positive events and a mean fluorescence intensity (MFI) of 6, 12 and 4 x 10^3^, respectively (Fig. 4C). The pattern of Rzv:TSP-2 when analysed by Western blot using anti-TSP-2 antiserum exhibits two bands (~ 30 and 60 kDa), indicating protein dimer formation. It is interesting to observe that BCG-Rzv:TSP-2 exhibits other two immunoreactive bands (between 30-45 kDa and above 60 kDa).

**Fig. 5:**
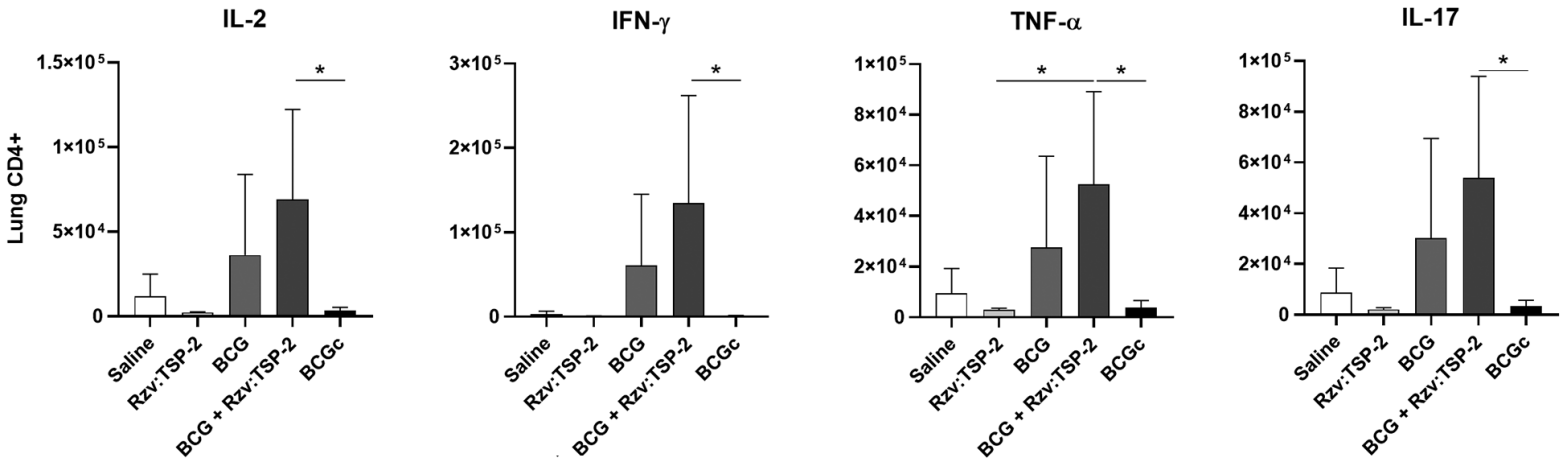
evaluation of immune response induced in immunised mice. Groups of mice (n = 5/group) were immunised with a single dose of saline, Rzv:TSP-2 (10 µg), bacillus Calmette-Guérin (BCG) [10^6^ colony-forming unit (CFU) ], BCG mixed with Rzv:TSP-2 or the coupled BCG-Rzv:TSP-2. Thirty days after, immune response was evaluated by measuring the phenotype of CD4^+^ T cells expressing TNF-α, IL-17, IFN-γ or IL-2 in the lungs.

*Immune response induced by BCG-Rzv:TSP-2 in immunised mice* - With the objective of evaluating the immunogenicity of BCG-Rzv:TSP-2 mice were immunised and the immune response evaluated 30 days later by immunophenotyping CD4^+^ and CD8^+^ T cells in the lungs and spleen. Unexpectedly, an increased antigen-specific response of lung CD4^+^ T cells was observed when mice were immunised with the mixture (BCG + Rzv:TSP-2) but not with BCG-Rzv:TSP-2 (Fig. 5). No statistically significant responses were observed for lung CD8^+^ T cells nor splenic CD4^+^ and CD8^+^ (not shown).

**Fig. 6:**
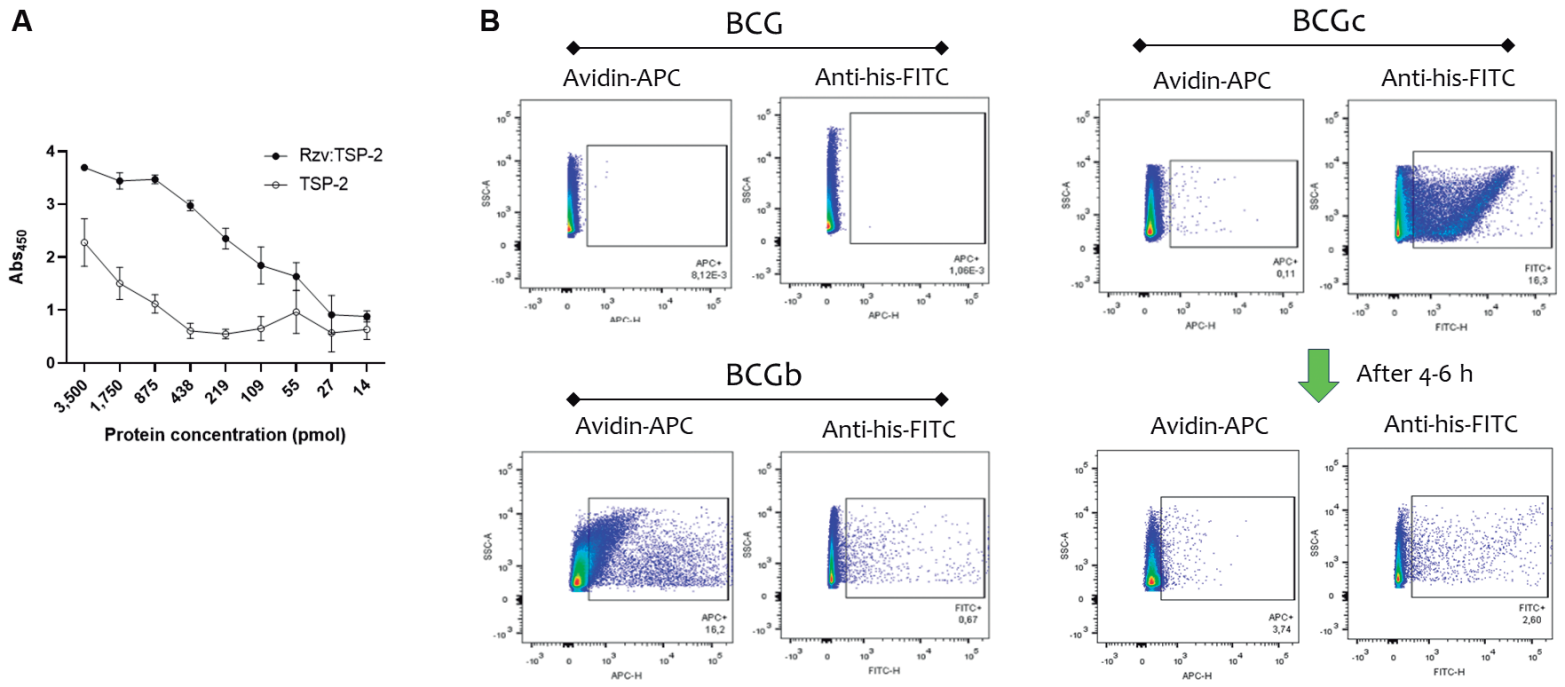
Rzv:TSP-2 binds to biotin. (A) Biotinylated protein was immobilised in enzyme-linked immunosorbent assay (ELISA) plates and the bind capacity of Rzv:TSP-2 evaluted by incubating serial dilutions of Rzv:TSP-2 in comparison to TSP-2. Reactivity was determined using anti-his-HRP and the reaction measured as Abs_450_. (B) Combined detection of biotin (using avidin-APC) and Rzv:TSP-2 (using anti-his-FITC) in the samples of bacillus Calmette-Guérin (BCG), biotinylated BCG (BCGb) or the coupled BCG-Rzv:TSP-2 (BCGc) right after antigen coupling or 4-6 h later.

We investigated the possible reasons for the low immunogenicity induced by BCG:Rzv-TSP-2. Although Rzv:TSP-2 demonstrated to functionally bind biotin-immobilised proteins (Fig. 6A) and the BCG’s surface (Fig. 6B) but the antigen bound to BCG demonstrated to be unstable (Fig. 6C).

## DISCUSSION

The continuous development of new and improved vaccine platforms enabled the generation of many new vaccines. We have previously demonstrated the suitability a system using outer membrane vesicles (OMVs) from *Neisseria lactâmica* as an immunogenic scaffold to *S. mansoni* antigens fused with rhizavidin. The OMVs coupled with Rzv:TSP-2 demonstrated to be highly immunogenic inducing 200 times the IgG antibody response in comparison to OMV mixed with Rzv:TSP-2.^19^ In an attempt to induce stronger cellular immune response in the lungs we sought to investigate whether this avidin-biotin affinity system would also be effective using BCG as scaffold.

In order to obtain a reasonable amount of antigen, the expression of Rzv:TSP-2 needed to be optimised. Also using TB as culture medium and Tunair full-baffled flasks, previous work reported the best conditions to be induction for 15 h at 23ºC (achieving a final OD_600_ ~ 15). This condition produced a final yield of 20 µg/mL of purified Rzv:TSP-2.^21^ We further improved the final yield after purification to ~ 170 µg/mL (~20 mL). It was interesting to note that solubility was similar in both conditions (induction at OD 0.6 and 3.0) while growth was higher in the later, as expected. While many other factors may influence the expression of a recombinant protein, time and temperature are two of the most important factors.^22^

We evaluated different biotinylation processes and reagents. The use of Amine-PEG_3_-Biotin requires reactive carboxylates (-COOH) which are produced by the use of EDC. EDC produces an intermediate form very unstable in water. Therefore, the inclusion of Sulfo-NHS can improve this reaction by creating a stable amine-reactive form. On the other hand, Sulfo-NHS-SS-Biotin reacts with primary amines (-NH2) of proteins. It does not require pre-activation with EDC and tends to not permeate through cell membranes.^23^ It is not known if it would also permeate through the complex mycobacterial cell wall and possibly affect BCG’s viability. The results suggested that the use of Amine-PEG_3_-Biotin may compromise the viability or BCG’s yield. It is uncertain whether the extra steps introduced by the use of EDC was the responsible for the lower viability/yield. On the other hand, biotinylation with Sulfo-NHS-SS-Biotin maintained the same viability as “untreated” BCG. Comparatively, the final yield of BCG was ~ 3 logs_10_ lower when using Amine-PEG_3_-Biotin.

The number of biotinylated events was similar among the conditions tested reaching ~ 20-30%. The sole study that describes the biotinylation of BCG report an efficiency of virtually 100% of the events.^24^ Even with optimisations of concentration of both reagent and bacteria per reaction, we achieved roughly 50% of the events biotinylated. Even with the possible differences (such as BCG strain, reagent concentration or the detection method used) this suggests that biotinylation of BCG is highly variable. For example, while Liao and cols suggest the use of 0.5 mM of Sulfo-NHS-SS-Biotin, in our results 8 µM produced the best results, a considerable difference in concentration.

After achieving a reasonable biotinylation reaction we investigate if an increasing amount of Rzv:TSP-2 would result in a higher number of BCGs coupled with the antigen and/or the intensity of the signal (amount of antigen/BCG). Varying the amount of Rzv:TSP-2 at 10, 100 and 700 µg/mL (the maximum concentration we achieved without precipitation of the recombinant protein) we observed a dose-response which resulted in 9, 30 and 37% of positive events. Interestingly, despite 700 µg/ml produced more positive events, the intensity of the signal (MFI) was higher when an intermediary concentration (100 µg/mL) of antigen was used. Another interesting observation was the two additional immunoreactive bands in the BCG-Rzv:TSP-2 that are absent in BCG only mixed with Rzv:TSP-2. This may occur due the strong bind (resistant to SDS in a reductive PAGE) of Rzv:TSP-2 to specific proteins in BCG’s surface. On this context, the main protein identified in the cell wall is HspX (~14 kDa).^25^ ^,^ ^26^ Predicted protein mass of Rzv:TSP-2 is 27 kDa, which sum would result in a 41 kDa protein. Quantification before and after incubation with BCGb revealed that the majority of the antigen’s total mass was still at the soluble phase indicating that only ~ 5-10% of the antigen was bound to BCG’s surface (not shown).

With the objective of evaluating if BCG-Rzv:TSP-2 would exert TSP-2-specific immune responses, mice models were immunised subcutaneously and the presence of T cells evaluated thirty days after. Unexpectedly, only BCG mixed with Rzv:TSP-2 induced specific CD4^+^ T cell responses in the lungs of the animals. The adjuvant effect and non-specific protective properties of the BCG vaccine against diseases other than tuberculosis are well-documented in the literature. This phenomenon may be applicable to what we’re observing in our results.^27^ ^,^ ^28^ ^,^ ^29^ The analysis of other cell types (CD8^+^ T cells) and systemic responses (in the spleens) revealed an overall poor immunogenicity (not shown). When we investigated the reasons for the poor immunogenicity, the protein Rzv:TSP-2 demonstrated to functionally bind biotin in immobilised biotin-containing proteins and also in BCG’s surface but, a few hours after coupled to BCG’s surface it appears to be rapidly degraded. Interestingly, neither avidin-APC bind the BCG after this indicating that the rhizavidin domain may have remained bound to biotin preventing avidin-APC bind but the antigenic portion (TSP-2) was degraded (or at least the C-terminal containing the hexahistidine tag). Another factor could be the quantification of BCGc, counting mycobacteria using CFU counts is a standard practice, but it’s quite a slow process. It can take anywhere from three to four weeks for colonies to become visible, especially depending on the strain of mycobacteria. Additionally, this method tends to underestimate bacterial numbers due to the formation of aggregates, making it less accurate, maybe polymerase chain reaction (PCR) could have been used as an alternative quantifying technique.^30^ ^,^ ^31^ As the process of biotinylation and antigen coupling require several washing steps, a considerable amount of bacterial mass is lost during this process and normalising the amount of BCGc for the immunisation is challenging.

In summary, this study presents the conditions to biotinylate BCG and couple rhizavidin-fusion antigens to its surface. While we demonstrate that antigen coupling is possible, stability issues are of concern and further improvements in both biotinylation and coupling reactions may be necessary to strengthen this strategy as a vaccine platform. If successful this could pave the way for its potential application to other diseases. We envision that several antigens of the same pathogen could be mixed altogether partially mimicking the surface-exposed targets of a specific pathogen. Even antigens from different strains/organisms could be blended aiming for a multivalent vaccine.
